# Efficacy, cost-utility and reach of an eHealth self-management application 'Oncokompas' that helps cancer survivors to obtain optimal supportive care: study protocol for a randomised controlled trial

**DOI:** 10.1186/s13063-017-1952-1

**Published:** 2017-05-22

**Authors:** Anja van der Hout, Cornelia F. van Uden-Kraan, Birgit I. Witte, Veerle M. H. Coupé, Femke Jansen, C. René Leemans, Pim Cuijpers, Lonneke V. van de Poll-Franse, Irma M. Verdonck-de Leeuw

**Affiliations:** 1 Department of Clinical Psychology, Amsterdam Public Health Institute, Vrije Universiteit Amsterdam, Amsterdam, The Netherlands; 2 EMGO+ Institute for Health and Care Research, Vrije Universiteit and VU University Medical Centre, Amsterdam, The Netherlands; 3 Cancer Centre Amsterdam (CCA), VU University Medical Centre, Amsterdam, The Netherlands; 4 Department of Epidemiology and Biostatistics, VU University Medical Centre, Amsterdam, The Netherlands; 5 Department of Otolaryngology–Head and Neck Surgery, VU University Medical Centre, Amsterdam, The Netherlands; 6 Department of Research, Netherlands Comprehensive Cancer Organisation, Eindhoven, The Netherlands; 7 Department of Medical Psychology, Tilburg University, Tilburg, The Netherlands; 8 Division of Psychosocial Research & Epidemiology, Department of Psychosocial Research, The Netherlands Cancer Institute, Amsterdam, The Netherlands

**Keywords:** Cancer survivors, eHealth application, Self-management, Patient activation, Supportive care

## Abstract

**Background:**

Cancer survivors have to deal with a wide range of physical symptoms, psychological, social and existential concerns, and lifestyle issues related to cancer and its treatment. Therefore, it is essential that they have access to optimal supportive care services. The eHealth self-management application Oncokompas was developed to support cancer survivors with where they need to turn to for advice and guidance, as well as to increase their knowledge on the availability of optimal support. A randomised controlled trial will be conducted to assess the efficacy, cost-utility and reach of Oncokompas as an eHealth self-management application compared with care as usual among cancer survivors.

**Methods/design:**

Adult cancer survivors diagnosed with breast, colorectal or head and neck cancer or lymphoma who are at 3 months to 5 years since curative treatment will be included. In total, 544 cancer survivors will be randomly assigned to the intervention group or a wait-list control group. The primary outcome measure is patient activation. Secondary outcome measures include self-efficacy, personal control, perceived patient-physician interaction, need for supportive care, mental adjustment to cancer and health-related quality of life. Furthermore, cost-utility outcomes will be assessed. *Reach* is defined as the percentage of cancer survivors who get access to Oncokompas within the context of this trial. Questionnaires will be administered at baseline, post-intervention and at 3- and 6-month follow-up.

**Discussion:**

In this study, we will evaluate the efficacy and cost-utility of Oncokompas among cancer survivors, as well as the reach of Oncokompas. These are essential first steps in the translation of research into practice and contribute to sustainable adoption, implementation and maintenance of an evidence-based Oncokompas.

**Trial registration:**

Netherlands Trial Register identifier: NTR5774. Registered on 8 March 2016.

**Electronic supplementary material:**

The online version of this article (doi:10.1186/s13063-017-1952-1) contains supplementary material, which is available to authorized users.

## Background

Cancer survivors have to deal with a wide range of physical symptoms, psychological, social and existential concerns, and lifestyle issues related to their cancer and its treatment. These problems can negatively affect health-related quality of life (HRQOL), may interfere with return to work and often result in higher medical care use [1, 2]. Therefore, it is essential that cancer survivors have access to optimal supportive care services. Supportive care for cancer survivors includes management of physical and psychological symptoms, social functioning, and existential and lifestyle issues related to cancer recurrence. Supportive care (e.g., physiotherapy, psychological support, support in the relationship with partner or children, support with existential questions or self-help interventions targeting a healthy lifestyle) is increasingly recognised as an integral part of quality cancer treatment [1, 2]. Although there is evidence that supportive care is effective [3–5], referral rates are low, and many cancer survivors have unmet needs [6, 7] related to, for example, fatigue, anxiety, depression or sexuality issues.

To improve accessibility to optimal supportive care services, cancer survivors are expected to adopt an active role in managing their own care [8]. Several studies have shown that self-management strategies ranging from educational interventions, exercise programs and (online) self-help interventions targeting psychological distress are beneficial for cancer survivors in terms of patient activation and self-efficacy [9–11]. Patient activation can be described as an individual’s knowledge, skill, and confidence for managing their health and healthcare [12]. Less activated people are more likely than highly activated patients to have unmet medical needs and to delay seeking medical care. As patients’ activation levels increase, they gain a greater sense of control over their health and feel empowered to take action [13].

There is growing interest in eHealth among patients, healthcare providers, healthcare assurance companies and policy-makers as a means to improve self-management [1]. To support cancer survivors in where they need to turn for advice and guidance, as well as increasing their knowledge on optimal support, the eHealth self-management application Oncokompas was developed. With Oncokompas, cancer survivors can monitor their quality of life by means of Patient-Reported Outcome Measures (PROMs), which is followed by automatically generated tailored feedback and personalised advice on supportive care services [14].

To ensure sustainable usage of Oncokompas, participatory design principles were followed [15], meaning that cancer survivors and healthcare professionals (HCPs) were involved in each step of the development process [14, 16, 17]. This approach resulted in an eHealth application which fits the needs of patients and HCPs. *See* the Methods section for more information on Oncokompas and its development process. The aim of the present study is to assess the efficacy and cost-utility of Oncokompas as an eHealth self-management application among cancer survivors, as well as the reach of Oncokompas within the context of this trial.

## Methods/design

This study is a randomised controlled trial (RCT) evaluating the efficacy and cost-utility of the eHealth application Oncokompas among cancer survivors, as well as the reach of Oncokompas. We closely followed the Standard Protocol Items: Recommendations for Interventional Trials (SPIRIT) checklist [18, 19] (*see* Additional file 1). Cancer survivors will be randomised into the intervention group (whose members will obtain access to the intervention) or a wait-list control group (whose members will obtain access to the intervention after a 6-month waiting period). The study is subdivided into two parts: part 1 concerns the reach and part 2 the efficacy and cost-utility of Oncokompas. The first part comprises the baseline assessment, and the second part comprises the post-intervention and follow-up assessments.

### Intervention

Oncokompas is an eHealth self-management application that supports cancer survivors in finding and obtaining optimal supportive care, adjusted to their personal health status and preferences. Oncokompas consists of three components: ‘Measure’, ‘Learn’, and ‘Act’. In the Measure component, cancer survivors can independently complete PROMs targeting the following quality-of-life domains: physical, psychological and social functioning, healthy lifestyle, and existential issues. Tumour-specific modules are available for patients with breast cancer, colorectal cancer, head and neck cancer, and lymphoma. Specific PROMs were selected by the project team in collaboration with teams of experts and on the basis of Dutch practical guidelines (from the Netherlands Comprehensive Cancer Organisation [IKNL]) and literature searches. Data derived from the Measure component are processed in real time and linked to tailored feedback to the cancer survivor in the Learn component. All algorithm calculations are based on available cut-off scores or are defined on the basis of Dutch practice guidelines, literature searches and/or consensus of teams of experts. In the Learn component, feedback is provided to the participant on the level of topics (e.g., depression, fatigue) by means of a three-color system: green (no elevated well-being risks), orange (elevated well-being risks) and red (seriously elevated well-being risks). Cancer survivors receive personalised information on the outcomes; for example, on the topic of depression, information is provided on the symptoms of depression and the proportion of cancer survivors who experience depressive symptoms. Special attention is paid to evidence-based associations between outcomes. For example, feedback on the association between depression and fatigue is provided if a participant has an orange or a red score on depression as well as fatigue. The feedback in the Learn component concludes with comprehensive self-care advice with tips and tools. All of this advice is tailored to the individual cancer survivor. In the Act component, cancer survivors are provided with personalised supportive care options based on their PROM scores and expressed preferences (e.g., preference for individual therapy versus group therapy). If a participant has elevated well-being risks (orange score), the feedback includes suggestions for self-help interventions. If a participant has seriously elevated well-being risks, the feedback includes advice to contact the participant’s own medical specialist or general practitioner [14, 17].

Several studies were conducted to optimally fit Oncokompas to patients’ and care providers’ preferences. Cancer survivors and HCPs were involved in each step of the development process. A needs assessment was conducted among cancer survivors and HCPs (step 1) [16]. Usability was tested by cancer survivors in two iterative cycles, and HCPs participated in cognitive walk-throughs (step 2) [17]. Cancer survivors participated in a multi-centre pilot study to assess feasibility (step 3) [14]. Oncokompas was optimised on the basis of the feasibility testing results.

### Study population

#### Part 1: inclusion and exclusion criteria

Inclusion criteria are cancer survivors diagnosed with breast, colorectal, or head and neck cancer or lymphoma; being aged ≥18 years (no upper limit); and having finished treatment with curative intent for 3 months to 5 years (all treatment modalities). Cancer survivors who have not yet completed endocrine therapy or immunotherapy for their breast cancer will be included 3 months to 5 years after their primary treatment. Exclusion criteria are male cancer survivors diagnosed with breast cancer and/or individuals with severe cognitive impairment, insufficient mastery of the Dutch language, and physical inability to complete a questionnaire.

#### Part 2: additional exclusion criterion

In addition to the inclusion and exclusion criteria of part 1, participants are excluded for part 2 if they do not have access to the Internet, do not use the Internet or do not have access to an email address.

### Study design

**Fig. 1:**
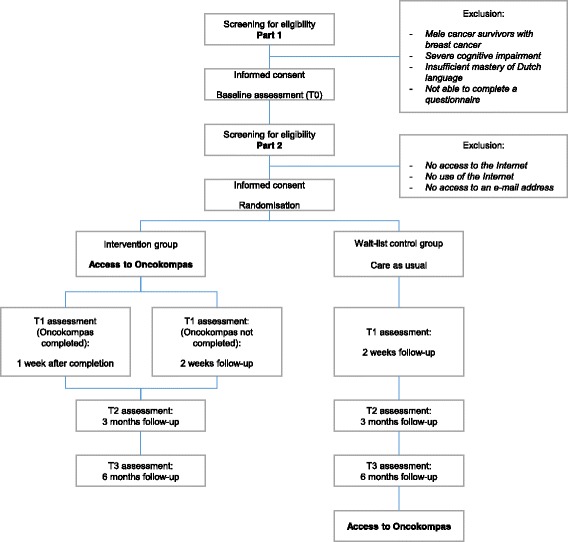
Flowchart of the randomised controlled trial

**Fig. 2:**
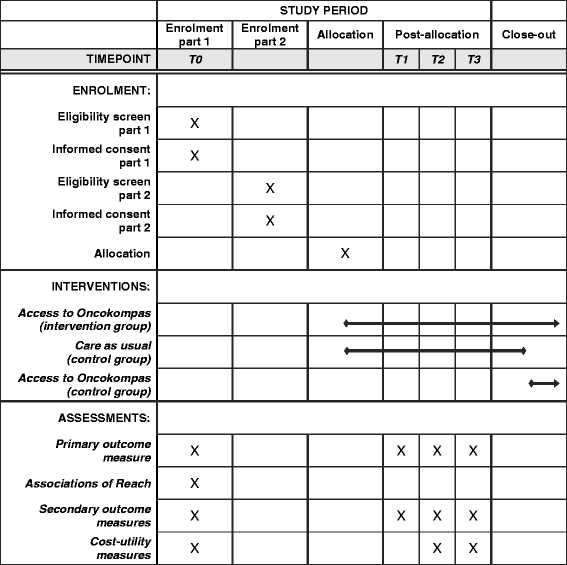
Standard Protocol Items: Recommendations for Interventional Trials (SPIRIT) schedule of enrolment, interventions and assessments

The study is introduced to eligible cancer survivors as a baseline study (part 1) and a follow-up study (part 2). Study information is given and informed consent is requested for both parts separately. Cancer survivors who fulfil the inclusion criteria and not the exclusion criteria for the first part are asked to participate in the baseline study. Baseline assessment (T0) will take place after the first informed consent form is signed. After completion of the baseline assessment, participants who fulfil the inclusion criteria and not the exclusion criteria for the second part are asked to participate in the follow-up study. After the second informed consent is given, participants will be randomly allocated to one of the two study arms. Follow-up assessments will take place post-intervention (T1) and at 3-month (T2) and 6-month (T3) follow-up. In the intervention group, T1 assessment takes place 1 week after completion of Oncokompas or 2 weeks after inclusion when Oncokompas is not completed. In the control group, T1 assessment takes place 2 weeks after inclusion. Participants allocated to the control group obtain access to Oncokompas after completion of the T3 assessment. A flowchart of the RCT is shown in Fig. 1, and the schedule of enrolment, interventions and assessments (according to SPIRIT guidelines) is provided in Fig. 2.

### Inclusion procedures

We will recruit cancer survivors through the Netherlands Cancer Registry (NCR), which is hosted by the IKNL. The NCR registers all newly diagnosed cancer patients within 6 months after diagnosis. Data collection will be performed using the registry of Patient Reported Outcomes Following Initial treatment and Long term Evaluation of Survivorship (PROFILES). PROFILES is a registry for the study of the physical and psychosocial impact of cancer and its treatment using a dynamic, growing, population-based cohort of both short- and long-term cancer survivors. PROFILES contains a large web-based component and is linked directly to clinical data from the NCR [20].

#### Part 1

A random sample of 1088 cancer survivors will be drawn from the NCR. This number is based on a power calculation (*see* ‘Sample size’ subheading) and an expected drop-out rate of 50% between parts 1 and 2. The selection of patients will be stratified by tumour type (breast, colorectal, and head and neck cancer or lymphoma) and time after finishing treatment (<6 months, 6–12 months, 12–24 months or 24–60 months after treatment). After excluding recently deceased patients, the (former) treating physicians are asked to verify the patients’ study eligibility (e.g., excluding patients with serious cognitive impairment or who are in transition to terminal care). Cancer survivors are invited to participate in the baseline study via a letter from their (former) treating physician. The letter includes a link to a secure website as well as a login name and password. Interested cancer survivors can log in and provide informed consent for the first part of the study and complete the baseline questionnaire. If a cancer survivor does not have access to Internet or prefers written rather than digital communication, an informed consent form and a paper-and-pencil questionnaire are sent by postal mail. Non-respondents will be sent a reminder letter and a paper-and-pencil questionnaire within 4 weeks. If they do not respond to this reminder, they will be contacted by telephone within 2 weeks.

#### Part 2

Cancer survivors who complete the baseline questionnaire will be invited to participate in the follow-up study. An email with information about the follow-up study and Oncokompas will be sent. Interested cancer survivors can provide informed consent for the second part of the study and complete the follow-up questionnaires on the same secure website where the baseline questionnaire resides. Cancer survivors who are not interested in participating in the study are asked about their reasons for non-participation. Non-respondents will be sent a reminder by email within 2 weeks. If they do not respond to this reminder, they will be contacted by telephone within 2 weeks.

### Randomisation

Cancer survivors who meet the inclusion criteria and give informed consent for the second part of the study are randomly allocated in a 1:1 ratio to either the intervention group (access to Oncokompas) or the wait-list control group (access to Oncokompas after a 6-month waiting period). Randomisation to either the intervention or the control group will be performed by a researcher not involved in the study using block randomisation. The blocks will have a length of 68. The researcher will determine all possible balanced combinations of assignment within the block (i.e., equal number for all groups within the block). Randomisation will be stratified by tumour type (breast, colorectal, and head and neck cancer or lymphoma). It is expected that this variable has prognostic relevance and therefore needs to be distributed evenly across both groups. The allocation sequence will be generated by PROFILES and will be made available by a data download from the PROFILES database. The researcher (AvdH) will assign participants either to the intervention group and invite participants to engage with Oncokompas by email or to the control group and place participants on the waiting list, where the participants’ email address is blocked from Oncokompas for 6 months.

### Outcome assessment

The primary outcome measure to assess efficacy of Oncokompas is patient activation. Secondary outcome measures include self-efficacy, personal control, perceived patient-physician interaction, mental adjustment to cancer, need for supportive care and HRQOL. Furthermore, cost-utility outcomes will be assessed. *Reach* is defined as the percentage of cancer survivors who get access to Oncokompas within the context of this RCT. To obtain insight into possible factors associated with reach, we will obtain data on socio-demographic and clinical characteristics, health literacy, health locus of control (HLC), Internet use, attitude towards eHealth and the outcome measures on efficacy.

Primary and secondary outcome measures to measure efficacy are collected at baseline, post-intervention and at 3- and 6-month follow-up. Cost-utility outcomes are collected at baseline and at 3- and 6-month follow-up. Outcome measures to investigate associations of reach are collected at baseline. An overview of the outcome measures is presented in Table 1.

**Table 1:** Study outcome measures and instruments

Outcome measure	Instrument
Efficacy^a^	
Primary outcome measure	
Patient activation	Patient Activation Measure (PAM)
Secondary outcome measures	
Self-efficacy	General Self-Efficacy Scale (GSE)
Personal control	Pearlin Mastery Scale (PMS)
Perceived patient-physician interaction	Five-item Perceived Efficacy in Patient-Physician Interactions (PEPPI-5)
Need for supportive care	34-item Short Form Supportive Care Needs Survey (SCNS-SF34) and head and neck cancer-specific module (SCNS-HNC)
Mental adjustment to cancer	Mental Adjustment to Cancer Scale (MAC)
Health-related quality of life	EORTC QLQ-C30
	EORTC QLQ-BR23
	EORTC QLQ-CR29
	EORTC QLQ-H&N43
	EORTC QLQ-HL27
	EORTC QLQ-NHL-LG20
	EORTC QLQ-NHL-HG29
Cost-utility measures^b^	
Outcome measures on cost-utility	
Quality-adjusted life-years	5 dimension EuroQol questionnaire (EQ-5D)
Medical costs	iMTA Medical Consumption Questionnaire (iMCQ)
Productivity costs	iMTA Productivity Cost Questionnaire (iPCQ)
Reach^c^	
Associations of reach	
Health literacy	Functional, Communicative and Critical Health Literacy scales (FCCHL)
Health locus of control	Multidimensional Health Locus of Control (MHLC)
Internet use	Adapted version of van de Poll-Franse and Van Eenbergen questionnaire [44]
Attitude towards eHealth	e-Health Impact Questionnaire (eHIQ)
Socio-demographic and clinical characteristics	Study-specific questionnaire

*Abbreviations: EORTC QLQ-C30/BR23/CR29/H&N43/HL27/NHL-HG29/NHL-LG20* 30-item core European Organisation for Research and Treatment of Cancer Quality of Life Questionnaire /breast cancer, 23 items/colorectal cancer, 29 items/head and neck cancer, 43 items/Hodgkin lymphoma, 27 items/non-Hodgkin lymphoma, high grade, 29 items/non-Hodgkin lymphoma, low grade, 20 items; *iMTA* Institute for Medical Technology Assessment

^a^Assessment at T0, T1, T2 and T3

^b^Assessment at T0, T2 and T3

^c^Assessment at T0

#### Efficacy

##### Primary outcome measure: patient activation

The Patient Activation Measure is a 13-item PROM on self-reported knowledge, skills and confidence in self-management of one’s health or chronic condition. Patients are asked to report their level of agreement with various statements on a 4-point Likert scale (i.e., strongly disagree, disagree, agree, strongly agree) or to indicate that the item is not applicable. A total score can be calculated by calculating a mean score of all the applicable items (items which were answered on the 4-point scale), which is transformed to a standardised activation score ranging from 0 to 100 [21].

##### Secondary outcome measures

*Self-efficacy*

The General Self-Efficacy Scale (GSE) is designed to assess optimistic self-beliefs regarding coping with a variety of difficult demands in life. The GSE consists of ten items scored on a 4-point Likert scale ranging from 1 (not at all true) to 4 (exactly true). The scores of the ten items are summed to give a total score. A higher score reflects a higher generalised sense of self-efficacy [22].

*Personal control*

The Pearlin Mastery Scale (PMS) measures global sense of personal control. It consists of seven items, and individuals respond to a 5-point Likert scale about the extent to which they agree (5 = strongly agree) or disagree (1 = strongly disagree) with the various statements. A PMS score ranges from 7 to 35, with a higher score reflecting greater mastery [23].

*Perceived patient-physician interaction*

The five-item Perceived Efficacy in Patient-Physician Interactions measures patients’ confidence in interacting with their main care provider using the short five-item version of the scale. Patients can indicate on a 5-point Likert scale (1 = not at all confident to 5 = completely confident) how confident they are that they, for example, know which questions to ask or are able to make the most out of their care provider visit [24, 25].

*Need for supportive care*

The 34-item Short Form Supportive Care Needs Survey (SCNS-SF34) measures the need and level of need for supportive care in the last month on the basis of 34 items using a 5-point, two-level response scale. The first response scale consists of two broad categories of need: ‘no need’ and ‘a need’. The ‘no need’ scale is further subdivided into ‘not applicable’ for issues that are not a problem to the patient and ‘satisfied’ for issues on which a patient needs support but the support is satisfactory. The ‘need’ category has three subcategories indicating the level of need for additional care: ‘low need’, ‘moderate need’ and ‘high need’ [26, 27].

In conjunction with SCNS-SF34, a tumour-specific module for patients with head and neck cancer can be used. The SCNS-HNC measures the need for supportive care concerning 11 HNC-specific issues using the same response scale as the SCNS-SF34 [28].

*Mental adjustment to cancer*

Cognitive and behavioural responses to cancer diagnosis and treatment are determined using the Mental Adjustment to Cancer scale (MAC). The MAC comprises five subscales: Fighting Spirit, Helplessness/Hopelessness, Anxious Preoccupation, Fatalism and Avoidance. The 40 items are rated on a 4-point Likert scale ranging from 1 for ‘definitely does not apply to me’ to 4 for ‘definitely applies to me’. A higher score represents a higher endorsement of the adjustment response [29].

*Health-related quality of life*

The 30-item core European Organisation for Research and Treatment of Cancer Quality of Life Questionnaire (EORTC QLQ-C30) is a cancer-specific quality-of-life questionnaire developed for repeated assessments within clinical trials. It was developed in a cross-cultural setting and is a valid and reliable instrument for quality-of-life assessments in various cancer populations. It contains five functional scales (physical, cognitive, emotional, social and role), a global quality-of-life scale, three symptom scales (pain, fatigue and nausea/vomiting) and six single items (dyspnoea, insomnia, loss of appetite, constipation, diarrhoea and financial difficulties). All scales and single items range in score from 0 to 100. A higher score on one of the functioning scales or the global quality-of-life scale represents a better quality of life, whereas a higher score on the symptom scales or the single items indicates a higher level of symptoms [30, 31].

In conjunction with the EORTC QLQ-C30, tumour-specific modules can be used. EORTC QLQ-BR23 is a module meant to be used among patients with breast cancer, varying in stage of disease and treatment. It consists of four functional scales (body image, sexual functioning, sexual enjoyment and future perspective), three symptom scales (systemic therapy side effects, breast symptoms and arm symptoms) and one symptom item (distress caused by hair loss) [32].

EORTC QLQ-CR29 is a module meant to be used among patients with colorectal cancer. It includes two functional scales (body image and future health perspective) and five symptom scales (micturition problems, gastrointestinal problems, defecation problems, sexual problems and chemotherapy-related problems) [33].

EORTC QLQ-H&N43 is a module meant to be used among patients with head and neck cancer. It contains 13 symptom scales (pain, swallowing, senses, speech, social eating, social contact, physical contact, skin, shoulder, body image, teeth, dry mouth and sticky saliva, and anxiety) and 6 symptom items (trismus, cough, lymphedema, wound healing, neurological problems and weight) [34].

EORTC QLQ-HL27, EORTC QLQ-NHL-LG20 and EORTC-QLQ-NHL-HG29 are modules meant to be used with patients with Hodgkin’s lymphoma, low-grade non-Hodgkin’s lymphoma and high-grade non-Hodgkin’s lymphoma, respectively. All modules have four multi-item scales, but they differ in the number of items per scale: symptom burden due to disease and/or treatment (4–7 items), physical condition/fatigue (4 or 5 items), emotional impact (4–6 items), and worries/fears health and functioning (8–11 items), with an extra item scale on neuropathy (2 items) for EORTC QLQ-NHL-HG29. For all scales, a higher score reflects worse or more symptoms/problems.

#### Outcome measures on cost-utility

A cost-utility analysis will be conducted; that is, the difference in total 6-month costs between the two arms will be compared with the difference in quality-adjusted life-years (QALYs) based on the 5-dimension EuroQol questionnaire (EQ-5D). The EQ-5D consists of five items measuring problems in five dimensions of quality of life (mobility, self-care, usual activities, pain/discomfort and anxiety/depression). Patients can answer that they have no problems, some problems or extreme problems [35]. The resulting profile of answers (1 of 243 possibilities) can be transformed to a value given by the general public: the EQ-5D index using the Dutch index tariff [36]. Furthermore, a visual analogue scale is included, which represents the patient’s judgment of his or her own health state on a scale from 0 (worst health state) to 100 (best health state).

Direct medical costs (healthcare and medication use), direct non-medical costs (travelling costs and help received from family or friends) and indirect non-medical costs (productivity losses) in the previous 3 months will be measured using an adapted version of the Institute for Medical Technology Assessment Medical Consumption Questionnaire (iMCQ) [37] and Institute for Medical Technology Assessment Productivity Cost Questionnaire (iPCQ) [38] of the Institute for Medical Technology Assessment (iMTA) of Erasmus University Rotterdam (Rotterdam, The Netherlands). In addition, a case report form on healthcare use in the hospital during the study period, including medical specialist visits, day treatment and hospital admission, will be completed using the hospital information system.

#### Reach

*Reach* is defined as the percentage of cancer survivors who get access to Oncokompas within the context of this RCT. More precisely, reach is the percentage of cancer survivors who are willing to participate in the second part of the study and thereby get access to Oncokompas (directly or after 6 months). For the numerator, cancer survivors who are willing to participate in the second part of the study and give their informed consent will be counted. For the denominator, all eligible cancer survivors who are invited to participate in the first part of the study will be counted.

Participants who complete the baseline questionnaire will be asked to participate in the follow-up study. To obtain insight into reasons for non-participation, participants not interested in the follow-up study will be asked to indicate their reasons for non-participation in the second part of the study (e.g., no interest in scientific research or no interest in the eHealth self-management application Oncokompas) by means of multiple-choice questions.

##### Associations of reach

To obtain insight into possible factors associated with reach, we will obtain data on socio-demographic and clinical characteristics, health literacy, HLC, Internet use, attitude towards eHealth, and the outcome measures on efficacy.

##### Socio-demographic and clinical characteristics

A study-specific questionnaire comprises questions about socio-demographics (age, marital status, family situation, education level) and clinical characteristics (co-morbidities). Clinical characteristics, including information on cancer type (breast, colorectal, head and neck cancer or lymphoma), cancer stage (TNM classification), cancer treatment and time since diagnosis, will be extracted from the NCR.

##### Health literacy

The validated Dutch translation of the self-report Functional, Communicative and Critical Health Literacy scales will be used to measure health literacy. The 14-item questionnaire asks for information on how often patients have had problems with health information and the extent to which they extracted, communicated and analysed health information. The answers are scored on a 4-point Likert scale ranging from 1 = ‘never’ to 4 = ‘often’ for functional health literacy and 1 = ‘easy’ to 4 = ‘rather difficult’ for communicative and critical health literacy [39, 40].

##### Health locus of control

HLC is measured with the Multidimensional Health Locus of Control (MHLC) scale form B. The MHLC scale comprises 18 diagnostic statements describing three dimensions of HLC: internal, powerful others and chance. The subscales ‘powerful others’ and ‘chance’ represent external HLC. People with high external HLC scores are presumed to have generalised expectancies that factors such as fate, luck, chance or powerful others will determine their health outcomes, whilst people with high internal HLC scores are presumed to hold the belief that someone becomes healthy or unwell as a result of their own behaviour. Each of the three subscales contains six items measured on a 6-point Likert scale ranging from 1 = ‘strongly disagree’ to 6 = ‘strongly agree’. The scores of each subscale range from 6 to 36 points; the higher the score, the stronger the self-perceived influence of a given factor [41–43].

##### Internet use

Internet use will be measured with an adapted version of the questionnaire developed by van de Poll-Franse and van Eenbergen [44]. It comprises three broad applications of Internet use (content, communication and community), of which only the application of ‘content’ will be used in this study, with ten multiple-choice items about the content of Internet use and content of Internet searches.

##### Attitude towards eHealth

General attitudes towards using the Internet to access health information will be measured using part 1 of the e-Health Impact Questionnaire, which consists of two subscales: attitudes towards online health information (five items) and attitudes towards sharing health experiences online (six items). All items have a 5-point response category ranging from ‘strongly disagree’ to ‘strongly agree’. Each scale will be transformed to a 0–100 metric, where 0 represents a low perceived value and 100 a high perceived benefit of using the Internet in relation to health [45].

### Sample size

To demonstrate presence of an effect between T3 and T0 of at least 0.5 standard units as statistically significant in a one-tailed test at α = 0.05 and a power of (1 − β) = 0.80, a minimum of 51 participants per arm in each condition will be required at follow-up. Anticipating a drop-out rate of 25% between T0 and T3, 68 participants per condition arm per tumour type need to be included at T0. The total study cohort thus comprises 544 cancer survivors representing 136 cancer survivors per tumour type (breast, colorectal, and head and neck cancer or lymphoma).

### Statistical analyses

Descriptive statistics will be generated for all socio-demographic and clinical characteristics and outcome measures. Chi-square tests, independent samples *t* tests (in case of normality of the measure) and Mann-Whitney *U* tests (in case of non-normality of the measure) will be used to analyse whether randomisation resulted in comparable patient groups. A *p* value of <0.05 will be considered significant. Analyses will be performed using IBM SPSS Statistics version 22 (IBM, Armonk, NY, USA) and Stata version 12.1 (StataCorp, College Station, TX, USA) software.

To investigate the efficacy of Oncokompas, linear mixed-effect models will be used to compare longitudinal changes in outcome measures for efficacy in both groups over time (intention-to-treat analyses). Independent samples *t* tests will be used to measure differences between the intervention and control groups at follow-up assessments. Cohen’s *d* [46] will be calculated as a measure of effect size (ES) for intervention group versus control group. Cohen’s *d* is computed as the difference between two means, divided by the pooled SD. The magnitude of the ES is classified as large (≥0.80), moderate (0.50–0.79) or small (<0.50) [47].

To investigate associations of the reach of Oncokompas within this RCT, chi-square tests, independent samples *t* tests and Mann-Whitney *U* tests will be used to analyse whether there are differences between participants and non-participants in the follow-up study (part 2) regarding baseline characteristics (part 1).

#### Cost-utility analyses

An incremental cost-utility ratio (ICUR) will be calculated to measure the cost per gained QALY. The ICUR will be calculated by dividing the incremental costs by the incremental QALYs using the formula: ICUR = (Costs_intervention_ − Costs_control_)/(QALY_intervention_ − QALY_control_). Total costs will be calculated using a societal perspective, including intervention costs, direct medical costs, direct non-medical costs and indirect non-medical costs. Direct medical and non-medical costs will be calculated by multiplying resource use by integral cost prices as presented in the Dutch Health Care Insurance Board (CVZ) guidelines on cost studies [48]. Indirect non-medical costs will be calculated using the friction cost approach as recommended in the CVZ guidelines [48]. The utility scores linked to the various health states of the EQ-5D [36] will be used to calculate QALYs by weighing the length of time spent in a particular health condition by the utility. Missing data on direct medical, direct non-medical and indirect non-medical costs measured using the cost questionnaire, and utilities measured using the EQ-5D will be imputed using multiple imputation. Because follow-up of the study is less than 1 year, neither costs nor effects will be discounted.

The uncertainty surrounding the ICUR will be assessed using bootstrapping with 5000 replications and projected on a cost-utility plane. In addition, cost-utility acceptability curves will be presented and sensitivity analyses will be performed, focusing on uncertainty around the most important cost parameters. The analysis will be conducted in accordance with the intention-to-treat principle.

## Discussion

In the proposed study, we will assess the efficacy and cost-utility of the eHealth self-management application Oncokompas among cancer survivors compared with care as usual, as well as the reach of Oncokompas within this trial. There is a growing need for interventions that meet cancer survivors’ supportive care needs in a personalised manner because referral rates to supportive care are low, whereas many have unmet needs [6, 7]. eHealth is proposed to be useful to improve access to and quality of care [49] and has a cost-saving potential [50]. The benefit of eHealth compared with care as usual is that eHealth may improve accessibility of supportive care without consulting HCPs, who have a tendency to inadequately refer patients for supportive care [51, 52]. An eHealth self-management application such as Oncokompas, which monitors patients’ quality of life, provides personalised advice and referral for supportive care services, could be a solution to meet cancer survivors’ individual supportive care needs by improving patient activation and self-efficacy [16]. Patients with high levels of activation understand their role in the care process, are more likely to engage in positive health behaviours, and are more likely to manage their health conditions more effectively. Less activated patients are more likely to have unmet needs [13].

By conducting this RCT, we will provide evidence on the efficacy of Oncokompas. In this way, we hope to establish whether access to an eHealth self-management application is effective in improving patient activation compared with care as usual. Secondary analyses will be conducted to investigate possible moderators that may influence the effect in order to gain knowledge on subgroups of cancer survivors who benefit the most from an eHealth self-management application such as Oncokompas. Also, mediation analyses will be conducted to elucidate whether the effect on patient activation is a direct effect of using Oncokompas or whether the effect is mediated by, for instance, improvement of mental adjustment to cancer.

Effects of self-management and eHealth interventions are often measured with so-called soft or patient-oriented outcome measures, because these types of interventions do not have pre-eminent outcomes like medical interventions [53]. Effects on patient-oriented outcome measures are relevant for patients themselves, but the clinical relevance of these effects is often unknown. In this study, efficacy is based on patient-oriented outcome measures; therefore, the (direct or indirect) effects of the use of Oncokompas on clinical outcomes will remain unknown.

It is argued that costs are often a major factor in determining whether a new intervention that is proven to be effective will be adopted, implemented or maintained [54]. Also, there is a need to explore whether it is possible to control healthcare costs while maintaining the quality of care [55]. Activated patients are expected to have better health outcomes and less healthcare use [56]. Because it is the aim of Oncokompas to improve self-management, it is expected that patients using Oncokompas will have less total costs (i.e., medical and non-medical costs) from a societal perspective compared with care as usual.

By investigating the representativeness and characteristics of cancer survivors who are willing to use Oncokompas in a study setting, we expect to be able to better reach the target population in the future. Usually, little is known about who is reached by eHealth interventions, whereas detailed information on non-participants is often not available or cannot be collected owing to ethical considerations [54]. Therefore, a two-step inclusion method was chosen for this RCT because in this way baseline characteristics (part 1) are available for non-participants in the follow-up study (part 2).

In this study, we are evaluating the efficacy, cost-utility and reach of Oncokompas among cancer survivors compared with care as usual. These are the first steps in the translation of research into practice [54] and might improve sustainable adoption, implementation and maintenance of an evidence-based Oncokompas.

### Trial status

The inclusion of patients for this study started in October 2016 and is ongoing.

## Additional file

Additional file 1: SPIRIT checklist. (PDF 167 kb)

## Abbreviations

CVZ: Dutch Health Care Insurance Board
eHIQ: e-Health Impact Questionnaire
EORTC QLQ-C30/BR23/CR29/H&N43/HL27/NHL-HG29/NHL-LG20: 30-item core European Organisation for Research and Treatment of Cancer Quality of Life Questionnaire /breast cancer, 23 items/colorectal cancer, 29 items/head and neck cancer, 43 items/Hodgkin lymphoma, 27 items/non-Hodgkin lymphoma, high grade, 29 items/non-Hodgkin lymphoma, low grade, 20 items
EQ-5D: 5-dimension EuroQol questionnaire
ES: Effect size
FCCHL: Functional, Communicative and Critical Health Literacy scales
GSE: General Self-Efficacy Scale
HCP: Healthcare professional
HLC: Health locus of control
HRQOL: Health-related quality of life
ICUR: Incremental cost-utility ratio
IKNL: Netherlands Comprehensive Cancer Organisation
iMCQ: Institute for Medical Technology Assessment Medical Consumption Questionnaire
iMTA: Institute for Medical Technology Assessment
iPCQ: iMTA Productivity Cost Questionnaire
MAC: Mental Adjustment to Cancer scale
MHLC: Multidimensional Health Locus of Control scale
NCR: Netherlands Cancer Registry
PAM: Patient Activation Measure
PEPPI-5: 5-item Perceived Efficacy in Patient-Physician Interactions
PMS: Pearlin Mastery Scale
PROFILES: Patient Reported Outcomes Following Initial treatment and Long term Evaluation of Survivorship
PROM: Patient-Reported Outcome Measure
QALY: Quality-adjusted life-year
RCT: Randomised controlled trial
SCNS-HNC: Supportive Care Needs Survey/head and neck cancer-specific module
SCNS-SF34: 34-item Short Form Supportive Care Needs Survey
SPIRIT: Standard Protocol Items: Recommendations for Interventional Trials

## References

[1] Aaronson NK, Mattioli V, Minton O, Weis J, Johansen C, Dalton SO (2014). Beyond treatment – psychosocial and behavioural issues in cancer survivorship research and practice. EJC Suppl.

[2] Fitch MI (2008). Supportive care framework. Can Oncol Nurs J.

[3] Speck RM, Courneya KS, Mâsse LC, Duval S, Schmitz KH (2010). An update of controlled physical activity trials in cancer survivors: a systematic review and meta-analysis. J Cancer Surviv.

[4] Hart SL, Hoyt MA, Diefenbach M, Anderson DR, Kilbourn KM, Craft LL (2012). Meta-analysis of efficacy of interventions for elevated depressive symptoms in adults diagnosed with cancer. J Natl Cancer Inst.

[5] Mewes JC, Steuten LMG, Ijzerman MJ, van Harten WH (2012). Effectiveness of multidimensional cancer survivor rehabilitation and cost-effectiveness of cancer rehabilitation in general: a systematic review. Oncologist.

[6] Boyes AW, Girgis A, D’Este C, Zucca AC (2012). Prevalence and correlates of cancer survivors’ supportive care needs 6 months after diagnosis: a population-based cross-sectional study. BMC Cancer.

[7] Harrison JD, Young JM, Price MA, Butow PN, Solomon MJ (2009). What are the unmet supportive care needs of people with cancer? A systematic review. Support Care Cancer.

[8] Jansen F, Van Uden-Kraan CF, Van Zwieten V, Witte BI, Verdonck-De Leeuw IM (2014). Cancer survivors’ perceived need for supportive care and their attitude towards self-management and eHealth. Support Care Cancer.

[9] Cimprich B, Janz NK, Northouse L, Wren PA, Given B, Given CW (2005). Taking charge: a self-management program for women following breast cancer treatment. Psychooncology.

[10] McCorkle R, Ercolano E, Lazenby M, Schulman-Green D, Schilling LS, Lorig K (2011). Self-management: enabling and empowering patients living with cancer as a chronic illness. CA Cancer J Clin.

[11] Kuijpers W, Groen WG, Aaronson NK, van Harten WH. A systematic review of web-based interventions for patient empowerment and physical activity in chronic diseases: relevance for cancer survivors. J Med Internet Res. 2013;15:e37. http://www.jmir.org/2013/2/e37/. 10.2196/jmir.2281 PMC3636300 23425685

[12] Hibbard JH, Mahoney ER, Stockard J, Tusler M (2005). Development and testing of a short form of the patient activation measure. Health Serv Res.

[13] Williams GC, McGregor H, Zeldman A, Freedman ZR, Deci EL, Elder D (2005). Promoting glycemic control through diabetes self-management: Evaluating a patient activation intervention. Patient Educ Couns.

[14] Duman-Lubberding S, van Uden-Kraan CF, Jansen F, Witte BI, van der Velden LA, Lacko M (2016). Feasibility of an eHealth application “OncoKompas” to improve personalized survivorship cancer care. Support Care Cancer.

[15] van Gemert-Pijnen JEWC, Nijland N, van Limburg M, Ossebaard HC, Kelders SM, Eysenbach G (2011). A holistic framework to improve the uptake and impact of eHealth technologies. J Med Internet Res.

[16] Lubberding S, van Uden-Kraan CF, Te Velde EA, Cuijpers P, Leemans CR, Verdonck-de Leeuw IM (2015). Improving access to supportive cancer care through an eHealth application: a qualitative needs assessment among cancer survivors. J Clin Nurs.

[17] Duman-Lubberding S, van Uden-Kraan CF, Peek N, Cuijpers P, Leemans CR, Verdonck-de Leeuw IM (2015). An eHealth application in head and neck cancer survivorship care: health care professionals’ perspectives. J Med Internet Res.

[18] Chan A-W, Tetzlaff JM, Altman DG, Laupacis A, Gøtzsche PC, Krleža-Jerić K (2013). SPIRIT 2013 statement: defining standard protocol items for clinical trials. Ann Intern Med.

[19] Chan AW, Tetzlaff JM, Gøtzsche PC, Altman DG, Mann H, Berlin JA, et al. SPIRIT 2013 explanation and elaboration: guidance for protocols of clinical trials. BMJ. J Med Internet Res. 2013;15(2):e37. 10.1136/bmj.e7586 PMC3541470 23303884

[20] van de Poll-Franse LV, Horevoorts N, van Eenbergen M, Denollet J, Roukema JA, Aaronson NK (2011). The patient reported outcomes following initial treatment and long term evaluation of survivorship registry: scope, rationale and design of an infrastructure for the study of physical and psychosocial outcomes in cancer survivorship cohorts. Eur J Cancer.

[21] Rademakers J, Nijman J, van der Hoek L, Heijmans M, Rijken M (2012). Measuring patient activation in The Netherlands: translation and validation of the American short form Patient Activation Measure (PAM13). BMC Public Health.

[22] Schwarzer R, Jerusalem M, Weinman J, Wright S, Johnston M (1995). Generalized Self-Efficacy scale. Causal control beliefs [Measures in health psychology: a user’s portfolio series].

[23] Pearlin LI, Schooler C (1978). The structure of coping. J Health Soc Behav.

[24] Maly RC, Frank JC, Marshall GN, DiMatteo MR, Reuben DB (1998). Perceived Efficacy in Patient-Physician Interactions (PEPPI): validation of an instrument in older persons. J Am Geriatr Soc.

[25] ten Klooster PM, Oostveen JCM, Zandbelt LC, Taal E, Drossaert CHC, Harmsen EJ (2012). Further validation of the 5-item Perceived Efficacy in Patient-Physician Interactions (PEPPI-5) scale in patients with osteoarthritis. Patient Educ Couns.

[26] Boyes A, Girgis A, Lecathelinais C (2009). Brief assessment of adult cancer patients’ perceived needs: development and validation of the 34-item Supportive Care Needs Survey (SCNS-SF34). J Eval Clin Pract.

[27] McElduff P, Boyes A, Zucca A, Girgis A (2004). Supportive care needs survey: a guide to administration, scoring and analysis.

[28] Jansen F, Witte BI, van Uden-Kraan CF, Braspenning AM, Leemans CR, Verdonck-de Leeuw IM (2016). The need for supportive care among head and neck cancer patients: psychometric assessment of the Dutch version of the Supportive Care Needs Survey Short-Form (SCNS-SF34) and the newly developed head and neck cancer module (SCNS-HNC). Support Care Cancer.

[29] Watson M, Homewood J (2008). Mental Adjustment to Cancer Scale: psychometric properties in a large cancer cohort. Psychooncology.

[30] Aaronson NK, Ahmedzai S, Bergman B, Bullinger M, Cull A, Duez NJ, Filiberti A, Flechtner H, Fleishman SB, de Haes JCJM, Kaasa S, Klee M, Osoba D, Razavi D, Rofe PB, Schraub S, Sneeuw K, Sullivan M, Takeda F. The European Organization for Research and Treatment of Cancer QLQ-C30: a quality-of-life instrument for use in international clinical trials in oncology. J Natl Cancer Inst. 1993;85:365–76. 10.1093/jnci/85.5.365 8433390

[31] Fayers P, Bottomley A (2002). Quality of life research within the EORTC—the EORTC QLQ-C30. Eur J Cancer.

[32] McLachlan SA, Devins GM, Goodwin PJ (1998). Validation of the European Organization for Research and Treatment of Cancer quality of life questionnaire (QLQ-C30) as a measure of psychosocial function in breast cancer patients. Eur J Cancer.

[33] Whistance RN, Conroy T, Chie W, Costantini A, Sezer O, Koller M (2009). Clinical and psychometric validation of the EORTC QLQ-CR29 questionnaire module to assess health-related quality of life in patients with colorectal cancer. Eur J Cancer.

[34] Singer S, Araújo C, Arraras JI, Baumann I, Boehm A, Brokstad Herlofson B (2015). Measuring quality of life in patients with head and neck cancer: update of the EORTC QLQ-H&N module, phase III. Head Neck.

[35] Brooks R (1996). EuroQol: the current state of play. Health Policy.

[36] Lamers LM, Stalmeier PFM, McDonnell J, Krabbe PFM, van Busschbach JJG (2005). Measuring the quality of life in economic evaluations: the Dutch EQ-5D tariff [in Dutch]. Ned Tijdschr Geneeskd.

[37] Bouwmans C, Hakkaart-van Roijen L, Koopmanschap M, Krol M, Severens H, Brouwer W (2013). Handleiding iMTA Medical Cost Questionnaire (iMCQ).

[38] Bouwmans C, Hakkaart-van Roijen L, Koopmanschap M, Krol M, Severens H, Brouwer W (2013). Handleiding iMTA Productivity Cost Questionnaire (iPCQ).

[39] Ishikawa H, Takeuchi T, Yano E (2008). Measuring functional, communicative, and critical health literacy among diabetic patients. Diabetes Care.

[40] van der Vaart R, Drossaert CHC, Taal E, ten Klooster PM, Hilderink-Koertshuis RTE, Klaase JM (2012). Validation of the Dutch functional, communicative and critical health literacy scales. Patient Educ Couns.

[41] Wallston K, Wallston B, DeVellis R (1978). Development of the Multidimensional Health Locus of Control (MHLC) Scales. Health Educ Monogr.

[42] Halfens R, Philipsen H (1988). A health specific control scale: validity and reliability of the MHLC [in Dutch]. Tijdschr Soc Gezondheidsz.

[43] Shneerson C, Taskila T, Greenfield S, Gale N (2015). A survey investigating the associations between self-management practices and quality of life in cancer survivors. Support Care Cancer.

[44] van de Poll-Franse LV, van Eenbergen MCHJ (2008). Internet use by cancer survivors: current use and future wishes. Support Care Cancer.

[45] Kelly L, Ziebland S, Jenkinson C (2015). Measuring the effects of online health information: scale validation for the e-Health Impact Questionnaire. Patient Educ Couns.

[46] Cohen J (1992). Statistical power analysis. Curr Dir Psychol Sci.

[47] Cohen J. Statistical power analysis for the behavioral sciences. 2nd ed. Hillsdale, NJ: Lawrence Erlbaum Associates; 1988. ISBN 0-8058-0283-5.

[48] Hakkaart-van Roijen L, Tan SS, Bouwmans-Frijters CAM (2011). Guidelines for pharmacoeconomic research in the Netherlands [in Dutch].

[49] Eysenbach G (2001). What is e-health?. J Med Internet Res.

[50] Bergmo TS (2015). How to measure costs and benefits of eHealth interventions: an overview of methods and frameworks. J Med Internet Res.

[51] Steginga SK, Campbell A, Ferguson M, Beeden A, Walls M, Cairns W (2008). Socio-demographic, psychosocial and attitudinal predictors of help seeking after cancer diagnosis. Psychooncology.

[52] Verdonck-de Leeuw IM, de Bree R, Keizer AL, Houffelaar T, Cuijpers P, van der Linden MH (2009). Computerized prospective screening for high levels of emotional distress in head and neck cancer patients and referral rate to psychosocial care. Oral Oncol.

[53] Baker TB, Gustafson DH, Shaw B, Hawkins R, Pingree S, Roberts L (2010). Relevance of CONSORT reporting criteria for research on eHealth interventions. Patient Educ Couns.

[54] Glasgow R, Vogt T, Boles S (1999). Evaluating the public health impact of health promotion interventions: the RE-AIM framework. Am J Public Health.

[55] Owens DK, Qaseem A, Chou R, Shekelle P, Clinical Guidelines Committee of the American College of Physicians (2011). High-value, cost-conscious health care: concepts for clinicians to evaluate the benefits, harms, and costs of medical interventions. Ann Intern Med.

[56] Hibbard JH, Mahoney ER, Stock R, Tusler M (2007). Do increases in patient activation result in improved self-management behaviors?. Health Serv Res.

